# Mechanistic Insights into CFTR Potentiation by the Antimicrobial Peptide Esc(1–21): Direct Interaction with the NBD1-NBD2 Interface

**DOI:** 10.1021/acsomega.6c03409

**Published:** 2026-08-17

**Authors:** Debora Baroni, Loretta Ferrera, Mario Passalacqua, Giacomo Cappella, Cecilia Astigiano, Y. Peter Di, Mattia Mori, Maria Luisa Mangoni

**Affiliations:** † Istituto di Biofisica, 117069 Consiglio Nazionale Delle Ricerche, CNR, via De Marini 6, Genova 16149, Italy; ‡ UOC Genetica Medica, IRCCS Istituto Giannina Gaslini, via Gerolamo Gaslini 5, Genova 16147, Italy; § Department of Experimental Medicine, Section of Biochemistry, 9302 University of Genova, Viale Benedetto XV 1, Genova 16132, Italy; ∥ Inter-University Centre for the Promotion of the 3Rs Principles in Teaching & Research (Centro 3R), Torino 10125, Italy; ⊥ Department of Biochemical Sciences, Laboratory Affiliated to Istituto Pasteur Italia-Fondazione Cenci Bolognetti, 9311 Sapienza University of Rome, Piazzale A. Moro 5, Roma 00185, Italy; # Center for Translational Science, 6614 Florida International University, 11350 SW Village Pkwy, Port St. Lucie, Florida 34987, United States; ∇ Department of Biotechnology, Chemistry and Pharmacy, 9313 University of Siena, via Aldo Moro 2, Siena 53100, Italy

## Abstract

Cystic fibrosis (CF) is a genetic disorder caused by variants in the gene encoding the CFTR ion channel, leading to defective epithelial chloride transport, dehydrated airway mucus, chronic infections, and progressive lung damage. Although CFTR modulators have improved clinical outcomes, a subset of patients remains unresponsive to current therapies, highlighting the need for alternative strategies. The frog skin-derived antimicrobial peptide Esc(1–21) was recently identified as a CFTR potentiator, likely through direct interaction with the interface of the channel’s cytosolic nucleotide-binding domains (NBDs); yet its molecular mechanism is poorly understood. Here, we engineered a cysteine-less CFTR scaffold by introducing cysteine pairs within or outside the predicted peptide-binding region and used molecular dynamics simulation to define the putative interaction site. Disulfide cross-linking assays performed in cells expressing these constructs validated the computational predictions and showed that the peptide selectively prevents cross-link formation only when cysteines are positioned at the NBD interface, thereby supporting a direct and specific interaction with CFTR. Importantly, confocal microscopy provided evidence for peptide intracellular internalization, together with colocalization with the ion channel, indicating that Esc(1–21) can access the cytosolic side of CFTR to enhance its activity. Overall, these findings (i) provide the first mechanistic insights into the interaction of Esc(1–21) with CFTR in a cellular context; (ii) identify the NBD1-NBD2 interface as a suitable target for peptide-based potentiators; and (iii) support the development of Esc(1–21) and its derivatives as novel multifunctional therapeutic candidates for CF treatment.

## Graphical abstract

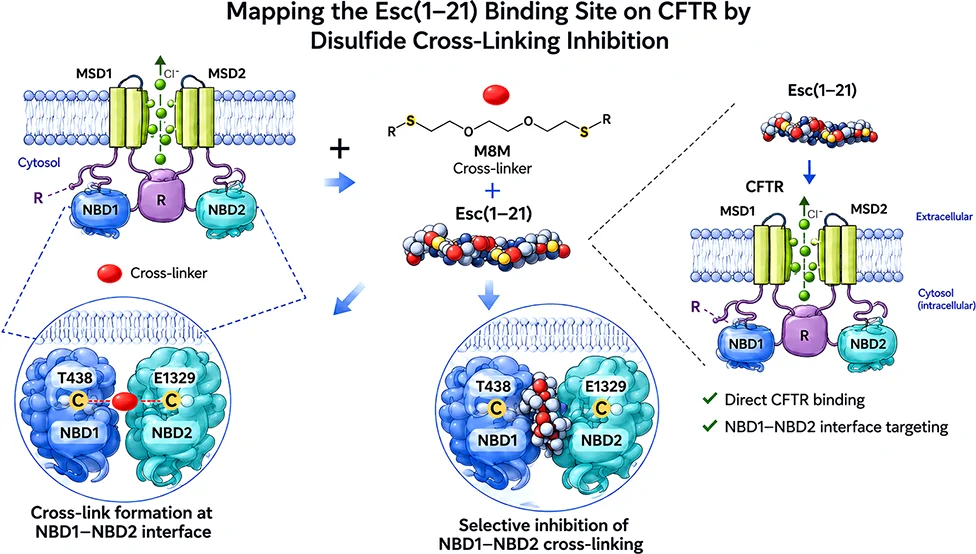

## Introduction

Cystic fibrosis (CF) is a severe autosomal recessive disease that primarily affects the lungs, caused by variants in the *CFTR* gene,^1^ which encodes a channel controlling the flow of chloride and bicarbonate ions through the apical membrane of epithelial cells.^2^ CFTR protein is composed of two transmembrane domains (TMDs), two cytosolic nucleotide-binding domains (NBDs), and a regulatory (R) domain.^3^ Channel activation involves phosphorylation of the R domain by cellular kinases, such as protein kinase A, PKA, and ATP-dependent NBD dimerization, to trigger the opening of the pore formed by its TMDs.^4,5^ The most common mutation, F508del, leads to a misfolded protein that is rapidly degraded, causing defective trafficking and impaired channel gating.^6,7^ Therefore, chloride secretion is reduced and sodium/water absorption increases, leading to thick, dehydrated airway mucus that promotes persistent lung infections, typically caused by *Pseudomonas aeruginosa*, with progressive tissue damage and pulmonary decline.^6^

CFTR variants are commonly classified into six functional categories according to their effects on channel protein synthesis, processing, gating, conductance, abundance, or stability.^7,8^ The most severe mutations are those belonging to Class I (e.g., G542X), resulting in a truncated, nonfunctional protein; Class II (e.g., F508del) affecting protein folding, trafficking, and gating; and Class III (e.g., G551D) hindering channel gating, often due to compromised NBD dimerization.^9^ Less severe variants encompass those of Class IV (e.g., R117H), which reduce ion conductance through the channel; Class V, characterized by a decreased amount of functional CFTR at the membrane level, due to an altered protein synthesis or aberrant splicing; and Class VI (e.g., S492F), which affect the stability of the protein at the cell surface.^8,10^ Recent advances in CF therapy have focused on small molecules, specifically CFTR modulators, to recover CFTR defects.^10–12^ Among these, “correctors” aid in proper folding and trafficking,^13^ while “potentiators” enhance channel gating.^14,15^ The triple combination therapy elexacaftor/tezacaftor/ivacaftor, consisting of the correctors elexacaftor and tezacaftor and the potentiator ivacaftor, has shown remarkable clinical benefits in patients carrying at least one F508del allele.^16^ However, these treatments remain ineffective or insufficient in subsets of patients, particularly those carrying variants associated with absence or minimal CFTR protein production, such as premature termination codons.^17^ Furthermore, current modulators lack intrinsic antimicrobial activity, an urgent unmet need in CF care, due to chronic and often multidrug-resistant pulmonary infections. In this context, previous work from our groups identified the antimicrobial peptide (AMP) Esc(1–21) GIFSKLAGKKIKNLLISGLKG-NH2 ^18,19^ derived from the N-terminal region of the frog-skin AMP esculentin-1a, and its diastereomer Esc(1–21)-1c containing d-Leu and d-Ser at positions 14 and 17, respectively.^20,21^ Both peptides (namely Esc peptides) showed potent membranolytic activity against the planktonic and biofilm forms of *P. aeruginosa* ^22^ and the capability to accelerate wound repair in bronchial epithelium.^20,23^ In murine lung infection models, low-dose intratracheal Esc(1–21)-1c reduced bacterial load by ∼2 logs, matching colistin efficacy^24^ and manifested an excellent safety profile without inducing inflammation or tissue injury.^25^ These peptides retain antimicrobial activity in CF-relevant conditions, such as acidic pH^26^ and mucus-rich environment as that found in both primary airway epithelial cells expressing F508del-CFTR, and animal models mimicking CF-lung (e.g., β-ENaC transgenic mice).^26^ Beyond their antimicrobial activity, these peptides were recently found to possess a novel CFTR potentiator function. They significantly increased chloride currents in cells expressing various CFTR mutants (F508del, G551D, G1349D), with an outcome comparable to that induced by the potentiator genistein.^27,28^ Previous inside-out patch-clamp experiments showed that Esc peptides increased ion currents controlled by F508del-CFTR in excised membrane patches exposed to ATP and PKA.^27^ This experimental configuration is compatible with a peptide action at the cytosolic side of CFTR but does not by itself define the detailed gating mechanism. Molecular dynamics (MD) simulations predicted that Esc(1–21) interacts with the lateral surface of NBD1, close to its interface with NBD2, through a network of hydrophobic and polar contacts.^27^ Based on these findings, a key question emerged: are Esc peptides internalized into cells and capable of accessing the cytoplasmic side of CFTR in a cellular system? Therefore, the present study aimed to further investigate the mechanism by which Esc peptides modulate CFTR function. Specifically, using Esc(1–21) as a reference peptide, we aimed to validate its interaction with the NBD domains of CFTR through biochemical disulfide cross-linking inhibition experiments complemented by MD simulations, and to assess peptide internalization in cells using fluorescence-based methods and confocal microscopy. Notably, these studies provided mechanistic insights into the mechanism underlying the interaction between Esc peptides and CFTR, paving the way for their advancement as CF therapeutics.

## Results

### MD Simulations of NBD Mutants

A common strategy to verify ligand binding to a protein pocket involves assessing the ability of the ligand to inhibit disulfide cross-linking between a cross-linker of proper length and two engineered cysteines (cys) introduced into the predicted binding region of the cys-less mutant protein.^29,30^ The inhibition occurs because if the ligand (peptide in our case) binds to this region, it sterically hinders the access of the cross-linker, precluding cross-linking formation.

Previous MD simulations on the NBD1–NBD2 heterodimer of F508del-CFTR indicated that Esc(1–21) predominantly interacts with NBD1, forming H-bond and hydrophobic contacts with residues Phe405, Phe409, Phe433, Thr438, Glu621 and Glu1329.^27,28^ Based on these predictions, cys residues were introduced at positions 438, 621, and 1329, generating three double cys-less mutants (Table 1): mutant 1 with T438C/E621C (both in NBD1), mutant 2 with T438C/E1329C (NBD1/NBD2), and mutant 3 with E1329C/V1379C (both in NBD2). In addition, two other mutants, mutant 4 with M607C/V1379C (NBD1/NBD2) and mutant 5 with S276C/Q1280C (TMD1/NBD2), containing one cys in TMD1 and another cys in NBD2, respectively, were generated and used as negative controls. The NBD heterodimer from cys-less/V510A wild-type CFTR (WT-CFTR) was also included as a reference.

**Table 1:** CFTR cys-Less Mutants and Affected Domains^a^

Mutant designation	Mutations	CFTR domain
**1**	T438C	**NBD1**
	E621C	**NBD1**
**2**	T438C	**NBD1**
	E1329C	**NBD2**
**3**	E1329C	**NBD2**
	V1379C	**NBD2**
**4**	M607C	**NBD1**
	V1379C	**NBD2**
**5**	S276C	**TMD1**
	Q1280C	**NBD2**

a Note that the V510A CFTR was selected for the generation of the cys-less mutants since V510A substitution facilitates maturation and processing of cys-less CFTR in cellular systems.^31^ Indeed, mutants in Table 1 were subsequently expressed in cells for cross-linking experiments to complement and validate the MD simulations.

In our study, MD simulations were initially employed as a predictive tool to guide the experimental strategy. Specifically, they were used to (i) assess whether the cys-less mutations (i.e., cys-less/V510A) altered the overall conformation of the NBD1/NBD2 heterodimer, (ii) predict the binding site of Esc(1–21) on the NBD1/NBD2 of T438C/E621C mutant (mutant 1, used as a positive control), and of M607C/V1379C mutant (mutant 4, used as a negative control); and (iii) evaluate the accessibility of the engineered cys residues in the presence of the peptide.

Prior to predicting the binding mode of Esc(1–21), MD simulations demonstrated that the cys-less/V510A CFTR retains the conformation of the NBD1/NBD2 heterodimer (Supporting Information, Figure S1).

MD simulations results supported the predicted binding mode of Esc(1–21) at the previously identified site of NBD1 at the interface with NBD2,^27,28^ in the selected NBD1/NBD2 cys-less mutants (Figure 1). Notably, in mutant 1, Esc(1–21) effectively shields the two cys residues (i.e., Cys438 and Cys621) from solvent exposure, likely preventing their interaction with a cross-linking agent (Figure 1A). In contrast, the Esc(1–21) binding site is preserved in the negative control (mutant 4), but the peptide does not mask the two target cys residues (i.e., Cys607 and Cys1379) which should therefore remain accessible to the cross-linking agent (Figure 1B).

**Figure 1:**
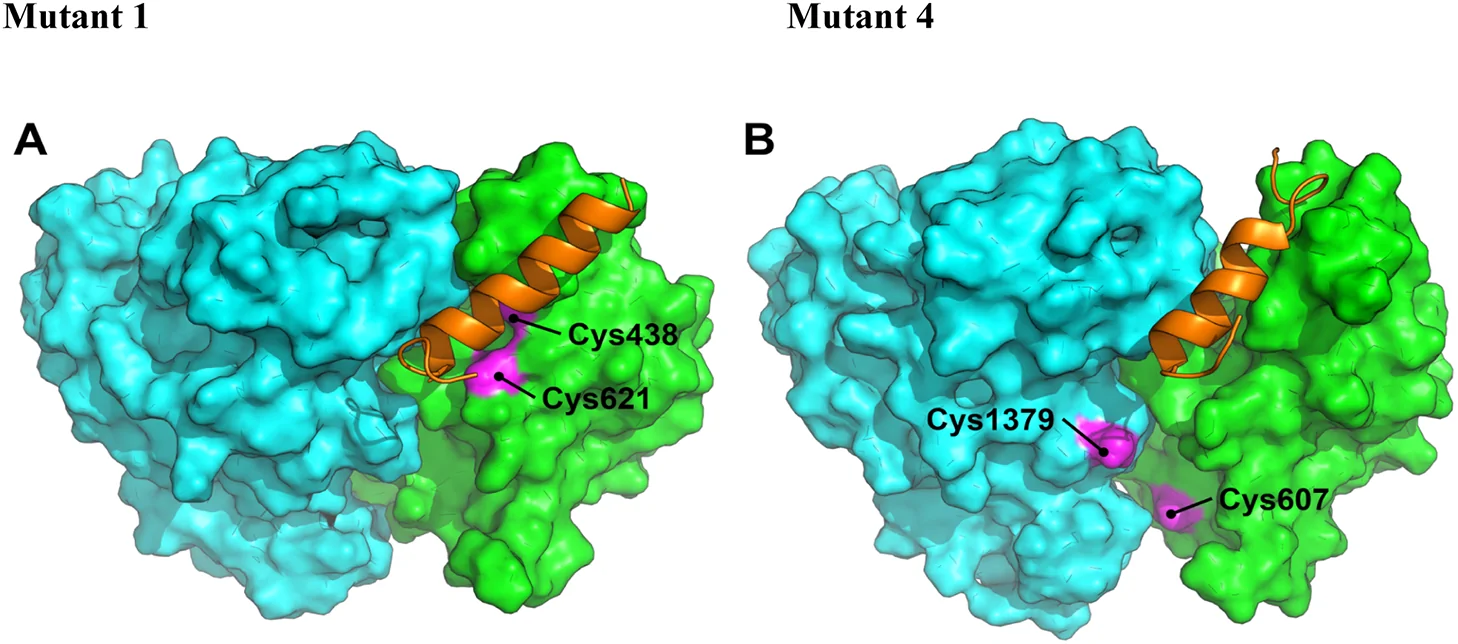
Binding mode of Esc(1–21) as predicted by MD simulations on the T438C/E621C (panel A) and M607C/V1379C (panel B) cys-less mutant NBD1/NBD2 heterodimer. The centroid frame of the most populated cluster of MD frames is shown; Esc(1–21) is colored orange, NBD1 is colored green, NBD2 is colored cyan. Cys residues are colored magenta and are labeled.

Overall, the MD simulation study provided a structural rationale for the design, cellular expression and experimental use of the corresponding CFTR mutants and supported the interpretation of biochemical cross-linking results discussed below.

### Electrophysiological Experiments in Cells Expressing WT-CFTR

Before generating CFTR mutants for biochemical cross-linking experiments, the effect of the peptide Esc(1–21) on the activity of the WT channel was evaluated. Consistent with previous studies,^27,28^ the transepithelial electrical conductance (TEEC) was measured in Fisher Rat Thyroid (FRT) cells expressing WT-CFTR without the addition of forskolin (FSK), to avoid maximal cAMP/PKA-dependent channel activation. CFTR-mediated TEEC was determined by transepithelial electrical resistance (TEER) measurements (see Materials and Methods) and calculated as the difference between the conductance recorded after a 10 min exposure of FRT epithelia to Esc(1–21) (2.5–20 μM) and that measured following CFTR inhibition. CFTR activity was therefore expressed as delta conductance, ΔG. As shown in Figure 2, all tested concentrations of the peptide elicited a significant increase in CFTR-dependent conductance within 10 min, indicating that Esc(1–21) can enhance CFTR activity under basal, non-FSK-stimulated conditions. To further assess whether the effect of Esc(1–21) on CFTR-dependent conductance could be related to activation of the cAMP pathway, intracellular cAMP levels were measured in FRT cells stably expressing WT-CFTR following acute peptide exposure. As expected, FSK markedly increased intracellular cAMP content compared with control cells (8.40 ± 2.00 vs 1.86 ± 0.30 pmol cAMP/10^6^ cells). In contrast, Esc(1–21) did not increase intracellular cAMP levels compared with control cells (1.79 ± 0.13 vs 1.86 ± 0.30 pmol cAMP/10^6^ cells). Moreover, the combination of Esc(1–21) with FSK did not further increase cAMP accumulation compared with FSK alone (8.22 ± 1.70 vs 8.40 ± 2.00 pmol cAMP/10^6^ cells). These results indicate that Esc(1–21) does not act through an FSK-like elevation of intracellular cAMP, supporting a mechanism distinct from upstream activation of the cAMP/PKA signaling pathway.^32^

**Figure 2:**
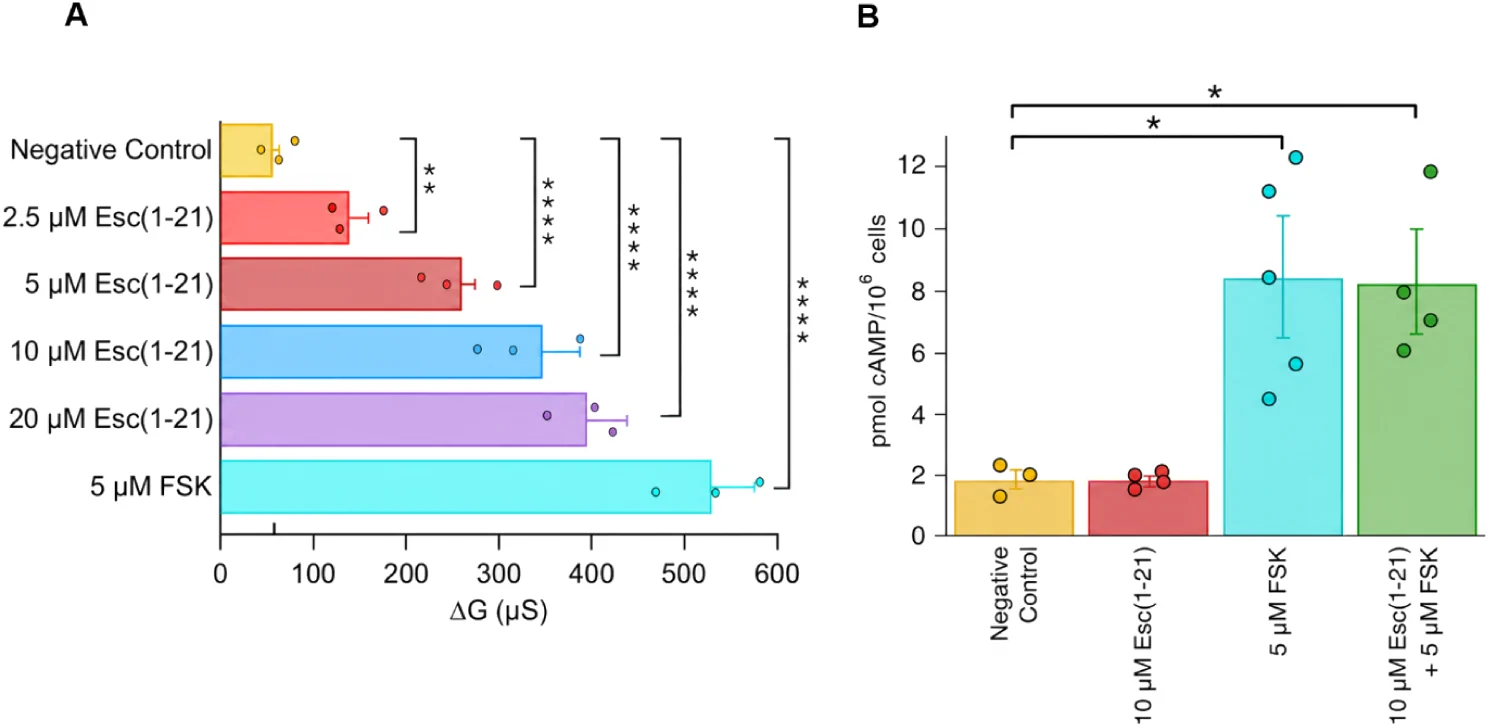
Effect of Esc(1–21) in FRT cells expressing WT-CFTR. Panel A: CFTR-mediated transepithelial conductance was assessed by TEER and calculated as the difference between conductance measured 10 min after the addition of 5 μM FSK alone (positive control) or peptide, at the indicated concentrations, and the conductance measured after CFTR inhibition (ΔG). Untreated cells were included as negative control. Panel B: Intracellular cAMP levels, expressed as pmol cAMP/10^6^ cells, measured after acute treatment with Esc(1–21) (10 μM), FSK (5 μM), or their combination. In both panels, bars represent mean ± SEM from at least three independent experiments; overlaid data points indicate individual measurements. Brackets indicate the groups compared in the statistical analysis which was assessed using a paired, two tailed Student’s *t* test and is indicated as follows: *p < 0.05, **p < 0.01, **** *p* < 0.0001.

### Cross-Linking Experiments

By using the cys-less/V510A WT-CFTR scaffold, the 5 double cys-less mutants (Table 1) were constructed and transiently transfected into human highly transfectable embryonic kidney 293 (HEK-t) cells. As reported in the literature,^31,33^ cross-linking between two cys residues introduced into the cys-less/V510A WT-CFTR scaffold, upon treatment with methanethiosulfonate cross-linkers causes CFTR to migrate more slowly on sodium dodecyl sulfate-polyacrylamide gel electrophoresis (SDS-PAGE) gels. This is evidenced in Western blots by the presence of an apparently higher molecular weight band (X-link) detected by the anti-CFTR antibody, together with the canonical B (immature, core glycosylated) and C (mature, fully glycosylated) CFTR bands.^34^ Panels B and C in Figure 3 show whole cell lysates from HEK-t cells either untransfected or transiently transfected with the T438C/E621C cys-less/V510A WT-CFTR plasmid (mutant 1) probed with antibodies against CFTR or actin. Both the short (M8M) and medium-sized (M11M) cross-linkers used in this study were effective in promoting cross-linking between the two cys residues introduced in this mutant, as evidenced by the presence of X-link bands in the lanes corresponding to lysates from HEK-t cells transfected with the mutant 1 plasmid and then treated with either the M8M or M11M cross-linkers. In contrast, Esc (1–21) at a concentration of 10 μM abolished cross-link formation between cys 438 and 621, as indicated by the disappearance of the X-link band (upper blots of panel B in Figure 3).

**Figure 3:**
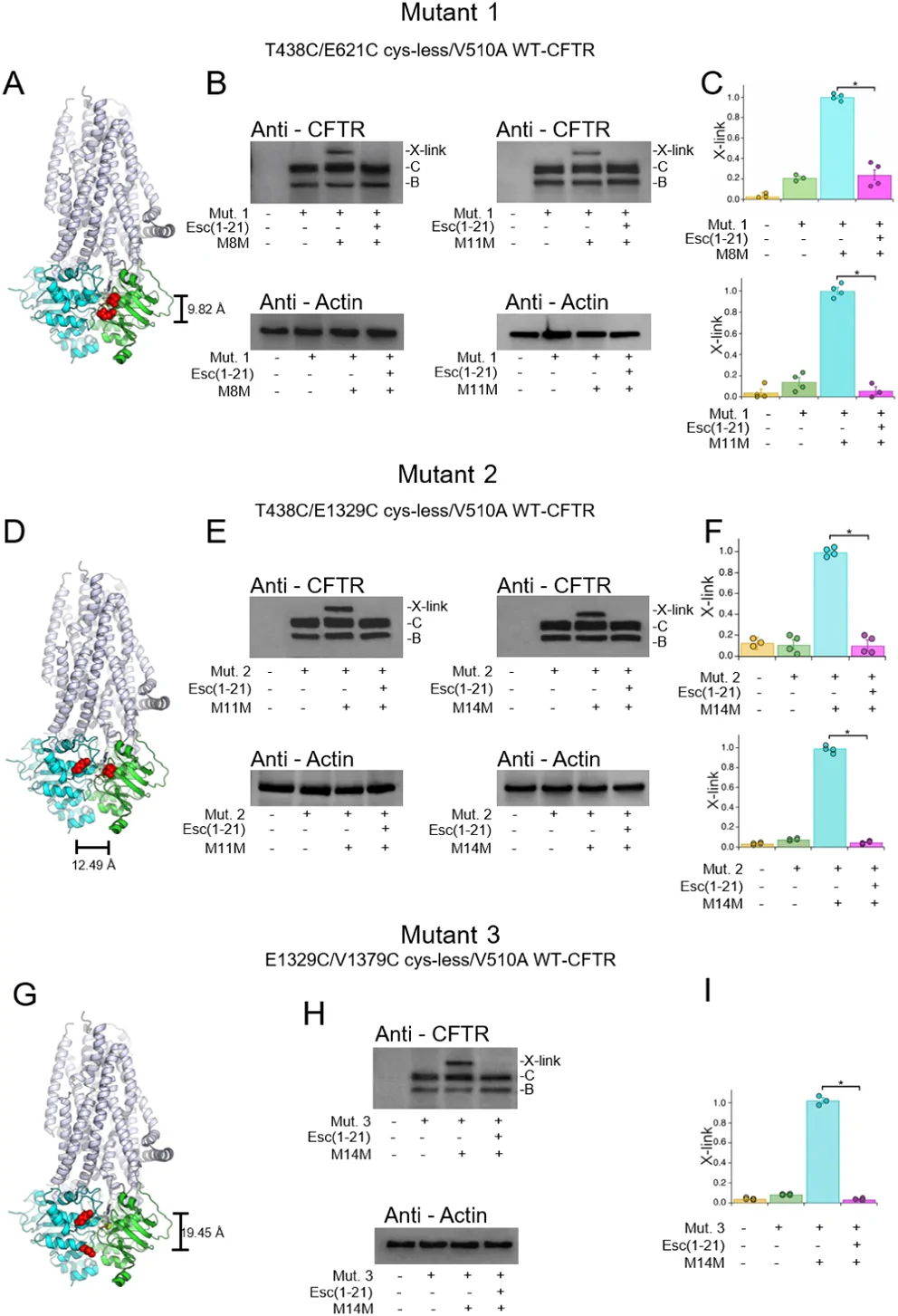
Effect of Esc(1–21) on disulfide cross-linking of CFTR double-cys mutants at the NBD1-NBD2 interface. Panels A, D, G: MD-based structural models of the cys-less/V510A WT-CFTR scaffold showing positions of the reintroduced cys residues in mutant 1 (T438C/E621C), mutant 2 (T438C/E1329C), and mutant 3 (E1329C/V1379C). Cys residues are shown in red, and median inter-residue distances are indicated. Panels B, E, H: HEK-t cells expressing the indicated mutants were incubated with (+) or without (−) 10 μM Esc(1–21) for 15 min and then treated (+) or not (−) with 0.1 mM M8M, M11M and M14M cross-linkers for 20 min. Lysates were analyzed by immunoblotting using anti-CFTR (upper blots) and anti-actin (lower blots) antibodies. Immature (B), mature (C) and cross-linked CFTR species (X-link) are indicated. Panels C, F, I: Bar graphs showing densitometric quantification of relative X-link abundance. Band intensities were normalized to actin and then expressed relative to the mean signal detected in the corresponding cross-linker-treated samples in the absence of Esc(1–21), which was set to 1. Bars represent mean ± SD from at least three independent experiments; overlaid data points indicate individual measurements. Statistical significance was assessed using a paired two-tailed Student’s *t* test comparing cross-linker-treated samples in the absence or presence of Esc(1–21). *p < 0.05.

Similarly, the two cys residues introduced at positions 438 and 1329 of the cys-less V510A WT-CFTR (mutant 2) were efficiently cross-linked by either the M11M or the M14M cross-linkers. Treatment with the Esc (1–21) peptide hampered cross-linking between the two cys (panels E-F in Figure 3).

Consistently, the M14M cross-linker induced cross-linking in CFTR mutant 3 (panels H–I in Figure 3), in which the cys residues are located at positions 1329 and 1379. Incubation with Esc(1–21) markedly reduced M14M-mediated cross-linking of this mutant (panels H and I in Figure 3), further supporting peptide occupancy of the predicted NBD1-NBD2 interface. Notably, in all these experiments, the choice of an appropriate cross-linker is crucial, as the length of its spacer arm must match the distance between the target cys residues. Accordingly, when the shorter cross-linker M11M was used for mutant 3, neither cross-linking nor an inhibitory effect was observed (Supplementary Figure S2). To confirm the selective binding of Esc(1–21) to the NBD1–NBD2 interface identified by computational studies and where residues 621, 438, and 1329 are located, two additional cys-less V510A WT-CFTR constructs (mutants 4 and 5) were examined. In these constructs, cys residues were reintroduced at positions outside the predicted binding pocket of the peptide and were therefore used as negative controls in cross-linking experiments.

In mutant 4, M11M efficiently promoted the cross-linking between the cys residues at position 607 and 1379; however, incubation with Esc (1–21) did not inhibit cross-link formation between these residues (panels B and C in Figure 4). A similar result was obtained for mutant 5. Cross-linking between cys residues at positions 276 and 1280 was observed after incubation with the M8M cross-linker, while no inhibition of cross-linking was detected in samples pretreated with the peptide prior to cross-linker addition (panels E and F in Figure 4).

**Figure 4:**
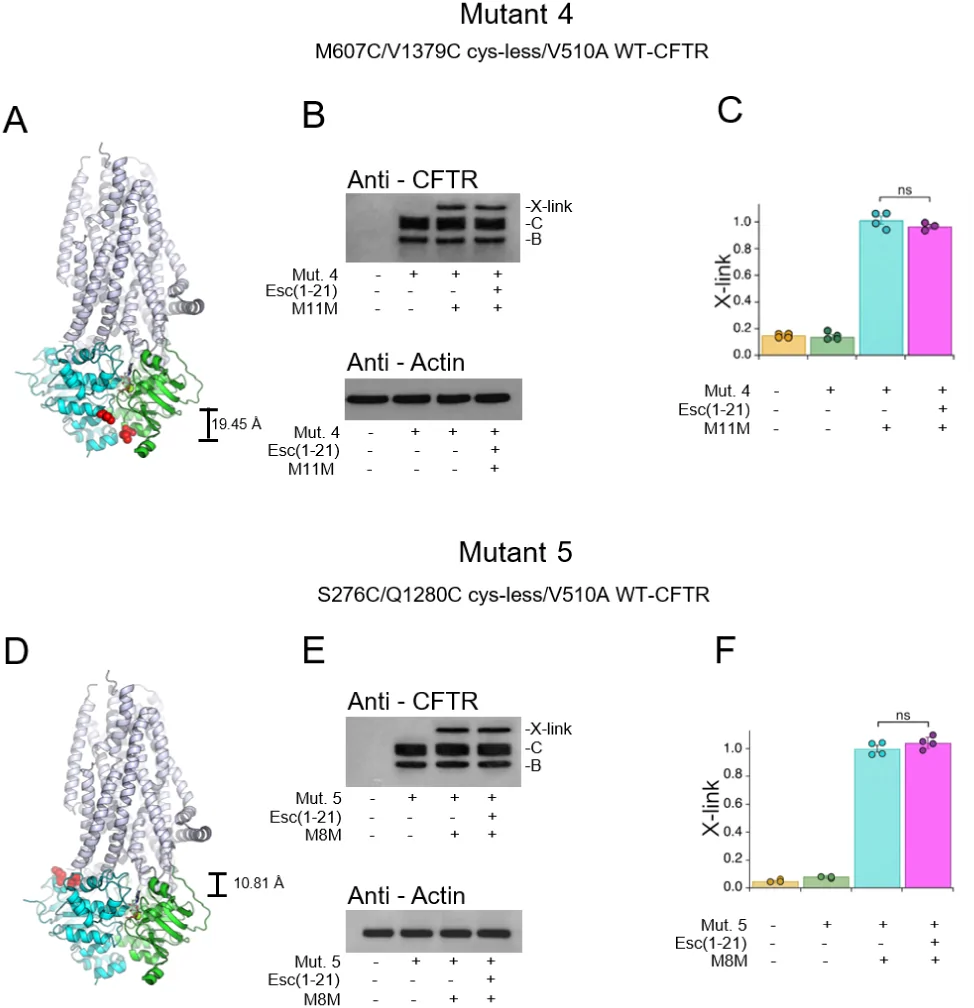
Effect of Esc(1–21) on cross-linking of CFTR double cys mutants outside the NBD1–NBD2 interface. Panels A, D: MD-based structural models of the cys-less/V510A WT-CFTR backbone showing positions of reintroduced cys residues in mutant 4 (M607C/V1379C) and mutant 5 (S276C/Q1280C); cys residues are in red and median inter-residue distance are indicated. Panels B, E: HEK-t cells expressing mutants were incubated with (+) or without (−) 10 μM Esc(1–21) for 15 min and then treated (+) or not (−) with 0.1 mM M11M or M8M cross-linkers for 20 min. Lysates were analyzed by immunoblotting using anti-CFTR (upper blots) and antiactin (lower blots) antibodies. Immature (B), mature (C), cross-linked CFTR species (X-link) are indicated. Panels C, F: Bar graphs showing densitometric quantification of relative X-link abundance. Band intensities were normalized to actin and then expressed relative to the mean signal detected in the corresponding cross-linker-treated samples in the absence of Esc(1–21), which was set to 1. Bars represent mean ± SD from at least three independent experiments; overlaid data points indicate individual measurements. Statistical significance was assessed using a paired two-tailed Student’s *t* test comparing cross-linker-treated samples in the absence or presence of Esc(1–21). No significant peptide-dependent reduction in X-link abundance was observed. ns, not significant.

### Confocal Microscopy

Confocal immunofluorescence microscopy was used to analyze the cellular distribution of the T438C/E621C cys-less/V510A WT-CFTR mutant (mutant 1), selected as a representative positive control, and of the Esc(1–21) peptide, in HEK-t cells (Figure 5). Transfected cells not exposed to the peptide and untransfected cells were also analyzed as controls (Figures S3–S5). Immunostaining revealed detectable enrichment of the cys-less mutant at the cell periphery, consistent with plasma membrane localization (panel A in Figures 5 and S3) although less pronounced than that observed for WT-CFTR (panel A in Figures 5 (inset) and S4). Quantitative analysis confirmed membrane localization of the mutant construct but revealed a significantly lower membrane-to-cytosol ratio (M/C ratio) compared to WT-CFTR (M/C ratio: 0.448 ± 0.024, n = 33 vs 0.828 ± 0.037, n = 35, panel A in Figure S6), in line with reduced plasma membrane localization of the cys-less CFTR.^35–37^ The distribution of Esc(1–21) peptide was then evaluated. In untransfected cells, incubated with Esc(1–21) (panel B in Figure S5), the peptide exhibited a predominantly intracellular distribution (M/C ratio: 0.38 ± 0.02, n = 38). In contrast, in CFTR-expressing cells, the peptide showed both peripheral and intracellular labeling (panel B in Figure 5). Quantitative analysis demonstrated increased peripheral enrichment of Esc(1–21) in cells expressing the cys-less mutant (M/C ratio: 0.51 ± 0.03, n = 31; compared to peptide-treated untransfected cells). A comparable increase was observed in WT-CFTR-expressing cells (0.58 ± 0.03, n = 31), with no statistically significant difference between cys-less and WT conditions (panel B in Figure S6). Merged images revealed a partial spatial overlap between CFTR and Esc(1–21), particularly at the plasma membrane (panel D in Figure 5). Co-localization analysis using Manders’ overlap coefficients confirmed a partial association between the two signals. In cells expressing the cys-less mutant, the fraction of CFTR signal overlapping Esc(1–21) (M1) was 0.252 ± 0.120, while the fraction of Esc(1–21) signal overlapping CFTR (M2) was 0.203 ± 0.091 (n = 34 cells). In WT-CFTR expressing cells (inset in panel D of Figure 5), Manders’ coefficients were M1 = 0.302 ± 0.192 and M2 = 0.290 ± 0.141 (n = 33 cells) indicating comparable degrees of spatial overlap between the two CFTR isoforms and the peptide.

**Figure 5:**
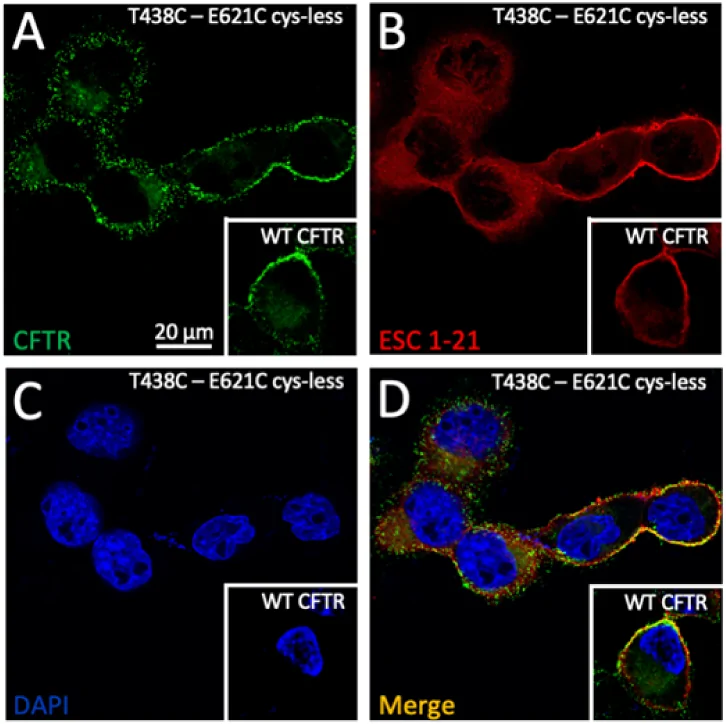
Confocal immunofluorescence analysis of colocalization between cys-less CFTR and the Esc(1–21) peptide. Representative confocal images of HEK-t cells expressing the T438C/E621C cys-less/V510A WT-CFTR construct following incubation with Esc(1–21). Panel A: CFTR cys-less mutant immunostaining (green) detected using a monoclonal anti-CFTR antibody and an Alexa Fluor 488-conjugated secondary antibody. Panel B: Esc(1–21) peptide localization (red), revealed using a rabbit polyclonal anti-peptide primary antibody and an Alexa Fluor 568-conjugated secondary antibody. Panel C: Nuclear counterstaining with 4′,6-diamidino-2-phenylindole (DAPI, blue). Panel D: Merged image highlighting CFTR cys-less-peptide colocalization (yellow). Insets show representative confocal images of cells expressing WT-CFTR incubated with Esc(1–21) peptide under the same experimental conditions. Co-localization was quantified using Manders’ overlap coefficients. The scale bar represents 20 μm.

Nuclear staining with DAPI confirmed cell integrity upon peptide treatment and facilitated identification of intracellular regions (panels C, D in Figure 5). Taken together, these results indicate that although the cys-less mutant shows reduced membrane enrichment compared to WT-CFTR, it retains the ability to promote peripheral recruitment of Esc(1–21), as reflected by similar levels of peptide membrane enrichment and comparable colocalization coefficients.

## Discussion

Defective anion transport through CFTR in the airways is known to underline mucus dehydration, lung infection, with gradual pulmonary tissue injury ultimately leading to compromised respiratory function.^11–13^ While CFTR modulators have substantially improved clinical outcomes, particularly in individuals receiving the triple combination therapy elexacaftor/tezacaftor/ivacaftor,^38^ approximately 10–15% of patients with CF are unresponsive to these therapies.^39^ Moreover, Ivacaftor only partially restores the activity of G551D-CFTR and, recent studies have demonstrated that dual-potentiator therapy can significantly enhance CFTR activity in gating mutations such as G551D.^40^ As a result, persistent pulmonary bacterial infections, especially in advanced stages of lung disease, remain a major therapeutic challenge and the identification of novel compounds that effectively address the fundamental defect of CF continues to be a priority.^41^

It is generally accepted that CFTR opens upon phosphorylation of the R domain and ATP-driven NBD dimerization, whereas it closes after ATP hydrolysis when the NBDs separate.^42^ Despite the precise coupling between NBD dimerization and gate movement within the TMDs of CFTR is under debate,^43^ any compound that enhances ATP binding, stabilizes NBD dimerization, and favors TMDs gate opening can function as a CFTR potentiator. Previous studies in our groups identified an unexpected property of the antimicrobial Esc peptides, thereby revealing a previously unrecognized biological activity of AMPs, namely their ability to restore CFTR function, although the underlying molecular mechanism has not been defined in detail.

MD simulations using the NBD heterodimer of F508del-CFTR previously predicted that Esc(1–21) interacts with the lateral surface of NBD1 near the NBD1–NBD2 interface, including Phe409, Glu621, Glu1329 and neighboring residues.^27^ This predicted interaction is consistent with a mechanism involving physical association of the peptide with the cytosolic region of CFTR; and this hypothesis is in line with prior inside-out patch-clamp experiments that suggested a cytosolic site of action for Esc(1–21).^27^ In addition, earlier studies pointed out the capability of Esc(1–21) to potentiate CFTR variants representative of class III mutations including G551D and G1349D.^28^ Computational modeling unveiled that the peptide interacts with the same NBD1–NBD2 interdomain pocket, supporting this region as an accessible target and druggable site for gating modulation across different dysfunctional CFTR forms.^28^ In this work, we investigated the molecular basis underlying Esc(1–21)-mediated potentiation of CFTR, focusing on its interaction with CFTR’s cytosolic domains. Using a cys-less/V510A CFTR scaffold, we engineered specific cys pairs at positions expected to lie within or outside the Esc(1–21) binding pocket. The selective inhibition of disulfide cross-linking observed in mutants carrying cys residues at sites 438/621, 438/1329 or 1329/1379, but not in mutants with cys located outside the predicted binding region, strengthens the interpretation that Esc­(1–21) can sterically interfere with cross-linker access when it occupies the selected NBD interface, corroborating the specificity of the peptide–CFTR interaction. A schematic summary of the predicted Esc(1–21)-binding region, including the main residues implicated by previous MD simulations and the cys pairs tested in the present study, is shown in Figure 6.

**Figure 6:**
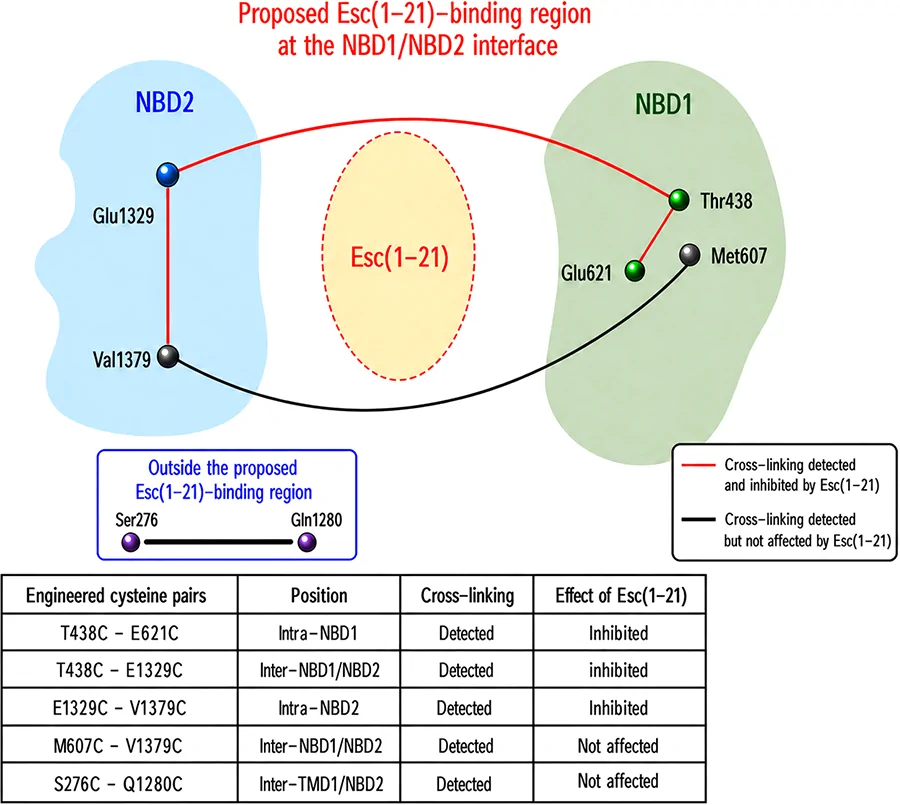
Schematic summary of the proposed Esc(1–21)-binding region at the CFTR NBD1/NBD2. interface. The scheme illustrates the predicted Esc(1–21) binding region located at the NBD1/NBD2 interface of CFTR and summarizes the outcomes of the cys cross-linking experiments used to probe this region. Engineered cys pairs within or adjacent to the proposed binding region are shown according to their domain location and their response to Esc(1–21). Red lines indicate cross-linking detected and inhibited by Esc(1–21), whereas black lines indicate cross-linking detected but not affected by the peptide. The distal control pair located outside the proposed binding region is shown separately. The table summarizes the position of each engineered cys pair, cross-linking detection, and the effect of Esc(1–21). The representation is schematic and not to scale.

MD analysis further confirmed that the cys-less/V510A retains the overall NBD1/NBD2 heterodimer architecture, supporting the use of these engineered constructs as reliable tools to probe ligand binding. Taken together, the computational and experimental data have provided converging evidence consistent with the interaction of Esc(1–21) with the cytosolic side of CFTR in a cellular context. The present MD simulations and cross-linking experiments with the engineered double-cysteine cys-less/V510A CFTR constructs should be viewed primarily as biochemical probes supporting peptide accessibility and occupancy at the predicted NBD1/NBD2 interface and providing spatial insight into the interaction between Esc(1–21) and CFTR, rather than a direct structural description of the gating transition. How binding of Esc(1–21) at this region is translated into increased CFTR activity remains to be determined by future electrophysiological, structural and CF-relevant epithelial model studies. Remarkably, former single-channel studies carried out by Mihályi and colleagues showed that spontaneous CFTR pore openings are coupled to tight NBD dimerization, indicating that coordinated NBD-pore movements are intrinsic to CFTR gating even in the absence of ATP.^44^ In this framework, Esc(1–21) may promote or stabilize NBD conformations permissive for channel opening. However, direct experimental validation under controlled phosphorylation and ATP conditions will be necessary to test this assumption.

In line with this mechanism, Esc(1–21) was found to be internalized into epithelial cells and to interact with CFTR channel. Using antibodies specifically raised against Esc(1–21), in combination with fluorophore-conjugated secondary antibodies and confocal microscopy, we supplied the first direct confirmation of cellular internalization of the native peptide. While prior studies reported the intracellular presence of Esc peptides in bronchial epithelial cells using rhodamine-conjugated derivatives, those approaches relied on chemically modified peptides. The current findings provide evidence that unmodified Esc(1–21) peptide can enter cells under the experimental conditions used, without causing any overt morphological alterations detectable by confocal microscopy, sustaining its ability to access the cytosolic face of CFTR, as also highlighted by the peptide-CFTR colocalization. Notably, quantitative imaging analysis revealed that Esc(1–21) has a predominantly intracellular distribution in untransfected cells, whereas expression of either WT-CFTR or the cys-less mutant significantly enhances peptide enrichment at the membrane level. This shift in membrane-to-cytosol ratio, together with the partial, but reproducible overlap between the fluorescence signals of CFTR variants and Esc(1–21), indicates that CFTR expression promotes peripheral accumulation of the Esc(1–21) and spatial overlap between the peptide and the channel, supporting preferential association with CFTR-expressing regions rather than a purely nonspecific membrane distribution. These findings further corroborate the hypothesis that Esc(1–21) is targeted to CFTR at the cytosolic interface, in agreement with the predicted binding site at the NBD1–NBD2 heterodimer. In parallel, functional assays highlighted that Esc(1–21) enhances CFTR-dependent conductance in epithelial cells expressing WT-CFTR under basal, non-FSK-stimulated conditions. This should not be considered as activation of fully unphosphorylated CFTR, because basal kinase activity in intact epithelial cells preparations is likely to maintain a fraction of CFTR in a phosphorylated or partially phosphorylated state, thereby sustaining residual channel activity.^43,45–47^ Since FSK activates CFTR through elevation of intracellular cAMP and subsequent PKA-dependent phosphorylation of the R domain, we further assessed whether Esc(1–21) increased cAMP levels under acute treatment conditions. As reported in Figure 2, Esc(1–21) did not raise intracellular cAMP content compared with control cells, implying that its effect on CFTR-dependent conductance does not depend on the increase in intracellular cAMP, such as that induced by FSK. Together with earlier inside-out patch-clamp data,^27^ these results are compatible with a direct action of Esc peptides at the cytosolic side of CFTR, although the detailed peptide-specific gating transition remains to be established. Although FRT cells expressing WT-CFTR do not reproduce the complexity of human airway epithelia, they provide a controlled epithelial model to assess acute CFTR-dependent conductance. The relevance of Esc peptides in CF-related contexts is supported by previous experiments showing that these peptides enhance CFTR function in both immortalized and primary human bronchial epithelial cells expressing disease-causing CFTR variants, including homozygous and heterozygous F508del-CFTR,^28^ likely upon intracellular entry.

Further studies in polarized primary human bronchial epithelial cells will be required to determine peptide uptake, apical accessibility and CFTR engagement in a more physiologically relevant epithelial context with mucus-producing phenotype.

It should be noted that most clinically advanced CFTR potentiators (e.g., ivacaftor/VX-770, GLPG1837) have been mapped to TMD regions.^48^ Cryo-EM directly visualized ivacaftor docked at a protein–lipid cleft formed by TM4/5/8, while photolabeling and docking studies identified nearby/overlapping sites for ivacaftor and GLPG1837,^49^ reflecting a TMD-centered allosteric mechanism for many small-molecule potentiators. Nevertheless, earlier work with genistein revealed that genetic variants at the NBD dimer interface significantly affect potentiator efficacy, underscoring the functional relevance of this region.^50,51^ Moreover, more recently, crotoxin-based peptides exerting a potentiator-like effect on channel activity have been reported to interact with the NBD1 domain of F508del-CFTR, supporting the notion that cytosolic CFTR domains are druggable and can be exploited for potentiation, particularly by larger ligands such as peptides.^52^ Therefore, our data with Esc(1–21) extend this concept and help to establish the NBDs as a suitable site for positioning an AMP with CFTR potentiator effect.

Although peptide-based strategies for CFTR modulation are less explored than small-molecules, a limited number of studies have pointed out that peptides can influence CFTR function through distinct mechanisms. For example, Hirsch and colleagues identified a cell-permeable PI3Kγ mimetic peptide capable of enhancing cAMP/PKA signaling in airways, thereby increasing CFTR phosphorylation and gating through an indirect, signaling-mediated mechanism rather than by direct interaction with the channel protein.^53,54^ Additional peptide-based approaches have focused on stabilizing CFTR at the plasma membrane. This is the case of peptides designed to inhibit the interaction between CFTR and the CFTR-associated ligand (CAL) containing PDZ-domain, ultimately prolonging the lifetime of CFTR at the cell surface and improving channel function once rescued to the membrane.^55^ Furthermore, the peptide SPX-101, derived from SPLUNC1 (BPIFA1), was found to indirectly enhance CFTR-mediated airways function in CF, by promoting epithelial sodium channel (ENaC) internalization, and thereby restoring airway surface hydration.^56^

Remarkably, the present study should be interpreted primarily as a mechanistic investigation of Esc(1–21)-CFTR interaction. The extent to which the interaction mechanism identified here contributes to the functional effects in CF-relevant epithelial models remains to be established in future studies.

## Conclusion

In conclusion, Esc peptides may represent a previously underexplored class of CFTR modulators with potential relevance to host–pathogen interaction in CF. Our data support a model in which the AMP Esc(1–21) can reach the intracellular compartment and occupy a region of CFTR close to the NBD1/NBD2 interface, thus promoting or stabilizing conformational states permissive for channel opening. This mechanism provides a coherent explanation for the peptide’s ability to potentiate both F508del-CFTR (once delivered to the plasma membrane) and class III gating mutants such as G551D and G1349D. These findings establish a mechanistic framework for future studies aimed at determining the contribution of this interaction to CFTR rescue and epithelial function in physiologically relevant disease models, while further exploring the capacity of Esc peptides to combine CFTR potentiation with antimicrobial and tissue-repair activities.

## Methods

### Cell Culture and Peptide

Human highly transfectable embryonic kidney 293 (HEK-t) cells were purchased from the Interlab Cell Line Collection (Genoa, Italy). Cells were grown in Dulbecco’s modified Eagle’s medium (DMEM) supplemented with 2 mM l-glutamine, 1% penicillin/streptomycin (100 U/mL), and 20% fetal bovine serum (FBS), at 37 °C and 5% CO_2_. For transfection, HEK-t cells were grown onto poly-l-lysine-coated 60 mm diameter culture dishes to 65% confluence in a complete medium. FRT cells with a stable expression of WT-CFTR were used for TEEC experiments.^57^ FRT cells were maintained at 37 °C in a 5% CO_2_ humidified atmosphere, in Coon’s modified Ham’s F-12 medium (Sigma-Aldrich, St. Louis, MO) supplemented with 2 mM l-glutamine, 10% FBS, 100 U/mL penicillin, and 100 μg/mL streptomycin. Esc(1–21) was purchased from Biomatik (Wilmington, USA). Peptide synthesis was performed using a stepwise solid-phase approach following the Fmoc strategy. The product was purified to a final purity of 98% by reverse-phase high-performance liquid chromatography, and its molecular mass was confirmed by mass spectrometry.

### TEEC Measurements to Evaluate CFTR Activity

To assess the effect of the Esc(1–21) peptide on the ion current regulated by CFTR, transepithelial conductance (TEEC) was measured in epithelial layers formed by FRT cells expressing WT-CFTR. Cells were seeded onto permeable supports (24-well Millicell inserts, PSHT01QR1) and maintained in their standard culture medium. The medium was then replaced with a saline solution containing (in mM): 130 NaCl, 2.7 KCl, 1.5 KH_2_PO_4_, 1 CaCl_2_, 0.5 MgCl_2_, 10 glucose, and 10 Na-Hepes (pH 7.4). This solution was added to both the apical and basolateral compartments of the permeable inserts.^58,59^ Initially, transepithelial resistance (TEER) was measured under basal conditions, without CFTR activation, using an epithelial voltmeter (MILLICELL ERS-2, Millipore, Burlington, MA). Afterward, either 5 μM FSK (used as a positive control) or various concentrations of the Esc(1–21) peptide (ranging from 2.5 to 20 μM) were applied to the apical side of the epithelium. TEER was recorded 10 min after compound addition, with samples kept at 37 °C. Finally, 30 μM PPQ102 was added apically to inhibit specifically CFTR activity, and TEER was measured again. TEER values obtained before and after CFTR inhibition were used to calculate the CFTR-dependent component of resistance for each experimental condition. All TEER measurements were then converted to TEEC using the formula TEEC = 1/TEER.^58^

### Intracellular cAMP Assay

FRT cells stably expressing WT-CFTR were seeded in 12-well plates at a density of 2 × 10^5^ cells/well and cultured for 24 h. Cells were rinsed with 1 mL Hanks’ Balanced Salt Solution (HBSS, Merck) and preincubated with the cAMP phosphodiesterase inhibitor Ro-20-1724 (10 μM, Merck) for 5 min at room temperature. Cells were then stimulated with Esc(1–21) (10 μM), FSK (5 μM), or their combination in HBSS for 15 min. After stimulation, supernatants were discarded and cells were lysed using the lysis buffer provided with the colorimetric competitive cAMP ELISA kit (ab234585, Abcam). Intracellular cAMP levels were measured according to the manufacturer’s instructions by reading absorbance at 405 nm using a microplate reader. Data are expressed as pmol cAMP/10^6^ cells and reported as mean ± SEM from three independent experiments.

### Construction of Mutants

Cys-less WT-CFTR was constructed by site-specific mutagenesis, replacing cys at positions 590 and 592 with leucine and the other 16 endogenous cys at positions 76, 126, 225, 276, 343, 491, 524, 657, 832, 866, 1344, 1355, 1395, 1400, 1410, and 1458 with alanine.^33^ Site directed mutagenesis was also used to introduce the V510A mutation that is known to promote the maturation of the cys-less WT-CFTR.^31^ As molecular docking analysis performed on Esc(1–21) identified residues at positions 438, 621, and 1329 within the peptide binding region,^27^ these residues were selected for conversion to cys and introduced in pairs into the cys-less/V510A WT-CFTR construct. Therefore, three double cys-less mutants, T438C (NBD1)/E621C (NBD1), T438C (NBD1)/E1329C (NBD2) and E1329C (NBD2)/V1379C (NBD2) were constructed to be used in cross-linking experiments. Two other constructs, M607C (NBD1)/V1379C (NBD2) and S276C (TMD1)/Q1280C (NBD2), containing pairs of cys residues located outside the predicted binding pocket of Esc(1–21), were generated as negative controls, using the same cys-less/V510A WT-CFTR scaffold. All cDNAs were sequenced to confirm the presence of mutations.

The distances between the couples of cys residues introduced into the cys-less/V510A WT-CFTR construct were calculated using the RASMOL software on the 6 msm .pdb structure of the CFTR protein, as well as confirmed by monitoring specific distances along MD trajectories.

### Treatments and Expressions of Mutants

HEK-t cells were transiently transfected using Lipofectamine 2000 (Invitrogen, Paisley, UK) with 4 μg of cDNA. The transfection medium (DMEM supplemented with 2 mM l-glutamine and without FBS) was replaced after 6 h with a fresh complete medium. To study the effect of Esc(1–21) on cross-linking, 48 h after transfection, cells were incubated either in the absence or in the presence of 10 μM Esc(1–21) for 15 min at 37 °C in a CO_2_ incubator. Cells were then harvested and incubated with 0.1 mM 3,6-dioxaoctane-1,8-diyl-bismethanethiosulfonate (M8M, 13 Å), 3,6,9-trioxaundecane-1,11-diyl-ismethanethiosulfonate (M11M, 16.9 Å) or 3,6,9,12-tetraoxatetradecane-1,14-diyl-bismethanethiosulfonate (M14M, 20.8 Å) (Selleck Chemicals, Cologne, Germany) for 20 min at 22 °C in the presence of 0.01% n-dodecyl-β-D-maltoside to enhance the solubility of the cross-linkers. The reactions were stopped by the addition of 2X SDS sample buffer containing 50 mM EDTA and no reducing agent. Samples (40 μL) were loaded on 7.5% to SDS-PAGE and an anti-CFTR mouse monoclonal (clone MM13–4, Millipore, dilution 1:200) antibody was used as primary antibody to probe Western blots. Goat antimouse horseradish peroxidase-conjugated antibody (dilution 1:2000; Santa Cruz Biotechnologies, Dallas, TX, USA) was used as a secondary antibody.

Immuno-detection was performed using Amersham ECL PLUS detection reagents (GE Healthcare Europe GmbH, Milan, Italy), and the images were captured using Amersham Hyperfilm ECL or visualizing them by using Uvitec chemiluminescence and epi-fluorescence imaging System (Cambridge, UK). To confirm the homogeneity of the loaded proteins, immunoblots were stripped by incubating them with stripping buffer (62.5 mM Tris–HCl, pH 6.8, 10% SDS, and 1% β-mercaptoethanol) for 30 min at 55 °C and reprobing them with an antiactin polyclonal antibody (1:2000, Sigma). Untransfected cell lysates were used as negative controls. All experiments were repeated at least three independent times.

### Immunocytochemistry and Confocal Microscopy

For immunocytochemical experiments, cells were plated on poly-l-lysine-coated glass coverslips (20 mm diameter) and transfected 6 h after plating with either WT-CFTR or the T438C (NBD1)/E621C (NBD1) cys-less/V510A WT-CFTR constructs; 48 h after transfection, cells were incubated with Esc(1–21) peptide (10 μM) for 15 min at 37 °C in a humidified CO_2_ incubator. In parallel, nontransfected cells were incubated with Esc(1–21) (10 μM, 15 min at 37 °C) under identical experimental conditions to evaluate peptide distribution in the absence of CFTR expression.

Cells were then fixed with paraformaldehyde, permeabilized with 0,1% Triton X-100, and blocked with 5% bovine serum albumin for 1 h at room temperature. After blocking, samples were incubated with the monoclonal anti-CFTR antibody and a rabbit polyclonal anti-peptide primary antibody,^60,61^ followed by Alexa Fluor–conjugated secondary antibodies, antimouse Alexa Fluor 488 (excitation 488 nm, emission 500–550 nm) and antirabbit Alexa Fluor 568 (excitation 561 nm, emission 580–620 nm, Thermo Fisher Scientific). Nuclei were counterstained with DAPI. Fluorescence images were acquired using a Nikon AX R confocal microscope equipped with a PLAN APO λD 60 × OFN25 DIC N2 NA 1.42 oil immersion objective (Nikon Europe B.V. Stroombaan 14, 1181 VX Amstelveen, The Netherlands). Excitation wavelengths/emission bandwidths were 488/500–540 for antimouse Alexa Fluor 488 and 561/580–630 for antirabbit Alexa Fluor 568. The pinhole size was set to 1 Airy Unit at a wavelength of 561 nm. Images were acquired with 2048 × 2048 pixels and 0.07 μm pixel size and sequential acquisition was performed to avoid cross-talk between color channels. All images used for quantitative analysis were acquired using identical laser power, detector gain, and offset settings within each experiment. Co-localization analysis between WT-CFTR or cys-less CFTR mutant and the Esc(1–21) peptide was performed on confocal images, and the degree of colocalization was quantified using Manders’ overlap coefficients.^62^ To assess subcellular distribution, membrane-to-cytosol fluorescence intensity ratios (M/C ratio) were determined for both WT-CFTR and T438C (NBD1)/E621C (NBD1) cys-less/V510A WT-CFTR (green channel) and Esc(1–21) (red channel). Quantitative analysis was performed in Fiji/ImageJ on original 16-bit images after uniform background subtraction when necessary. For each cell, a region of interest (ROI) outlining the entire cell was manually defined. A membrane ring region was generated by enlarging and shrinking the original ROI by 2 pixels and applying an XOR operation to isolate the peripheral region. A cytosolic ROI was drawn within the intracellular area, avoiding nuclear regions and membrane borders. Mean fluorescence intensity was measured in both regions, and the M/C ratio was calculated as M/C ratio = Mean membrane intensity/Mean cytosolic intensity. At least 30 cells per condition were analyzed.

### MD Simulations

MD simulations were run with AMBER24, using the protocol validated in previous works.^63,64^ Briefly, simulation systems were built by placing the NBD1/NBD2 heterodimer in a rectilinear simulation box of TIP3P-type water molecules. Esc(1–21) was placed in the simulation box at a distance higher than 40 Å from the NBDs, with a random orientation and in a random position. Na^+^ counterions were added up to system neutrality. Initially, the solvent was energy minimized by 500 cycles with the steepest descent algorithm (SD) followed by 2500 cycles with the conjugate gradient algorithm (CG) while keeping the solute as frozen. Then, the solvated solute was energy minimized for 1000 cycles with the SD, and subsequent 5000 cycles with the CG. After energy minimization, systems were heated to 300 K at constant volume with the Langevin thermostat for 1 ns; density was further equilibrated for 1 ns at constant pressure with the Berendsen barostat, before a preliminary MD trajectory was produced for 50 ns. Finally, MD trajectories were produced for 500 ns at constant pressure without positional restraints. The ff14SB force field was used; time-step was set at 2 fs. Analysis of MD trajectories was carried out with the CPPTRAJ software.^65^

### Statistical Analysis

Quantitative data from independent experiments are reported as mean ± standard deviation (SD) for biochemical assays and Western blot densitometry, and as mean ± standard error of the mean (SEM) for single-cell confocal microscopy and TEEC experiments. Statistical analyses were performed using Igor Pro 9 (WaveMetrics, USA). Comparisons between two groups were conducted using Student’s *t* test, while confocal microscopy data were analyzed using the Kruskal–Wallis one-way nonparametric ANOVA followed by Dunn’s multiple comparisons test. A p-value <0.05 was considered statistically significant.

## Supplementary Material



## Data Availability

Data reported in this work is available upon request to the corresponding authors.

## Notes

Structural comparison of the MD representative structure describing the wild-type NBD1/NBD2 heterodimer (Figure S1); effect of Esc(1–21) on disulfide cross-linking of the CFTR double cys-less mutant 3 at the NBD1-NBD2 interface (Figure S2); confocal images of HEK-t cells expressing the T438C/E621C cys-less/V510A WT-CFTR construct (Figure S3); confocal immunofluorescence analysis of WT-CFTR (Figure S4); confocal immunofluorescence analysis of Esc(1–21) in untransfected HEK-t cells (Figure S5); quantification of membrane-to-cytosol fluorescence intensity ratios (M/C ratio) (Figure S6) (PDF)
