# Supplementary material for: Mechanistic Insights into CFTR Potentiation by the Antimicrobial Peptide Esc(1–21): Direct Interaction with the NBD1-NBD2 Interface
Source: ACS Omega. 2026 Aug 17;11(34):51094–105. doi: 10.1021/acsomega.6c03409 (PMC13625118; doi:10.1021/acsomega.6c03409)
Supplement: Supplementary file 1 [file ao6c03409_si_001.pdf]

SUPPLEMENTARY INFORMATION

Mechanistic insights into CFTR potentiation by the antimicrobial peptide Esc(1-21): direct interaction with the NBD1-NBD2 interface

Debora Baroni[a]*, Loretta Ferrera[b], Mario Passalacqua[c,d], Giacomo Cappella[e], Cecilia Astigiano[c], Y. Peter Di[f], Mattia Mori[g], Maria Luisa Mangoni[e]*

.

[a]Istituto di Biofisica, Consiglio Nazionale delle Ricerche, CNR, Via De Marini 6, 16149 Genova, Italy; [b]UOC Genetica Medica, IRCCS Istituto Giannina Gaslini, Via Gerolamo Gaslini 5, 16147 Genova, Italy; [c]Department of Experimental Medicine, Section of Biochemistry, University of Genova, Viale Benedetto XV 1, 16132 Genova, Italy; [d]Inter-University Centre for the Promotion of the 3Rs Principles in Teaching & Research (Centro 3R), 10125 Torino, Italy; [e]Department of Biochemical Sciences, Laboratory Affiliated to Istituto Pasteur Italia-Fondazione Cenci Bolognetti, Sapienza University of Rome, Piazzale A. Moro, 5, 00185 Roma, Italy; [f]Center for Translational Science, Florida International University, 11350 SW Village Pkwy, Port St. Lucie, Florida, USA; [g]Department of Biotechnology, Chemistry and Pharmacy, University of Siena, Via Aldo Moro 2, 53100 Siena, Italy

* *Correspondence to:* Debora Baroni (debora.baroni@ibf.cnr.it) and Maria Luisa Mangoni (marialuisa.mangoni@uniroma1.it)

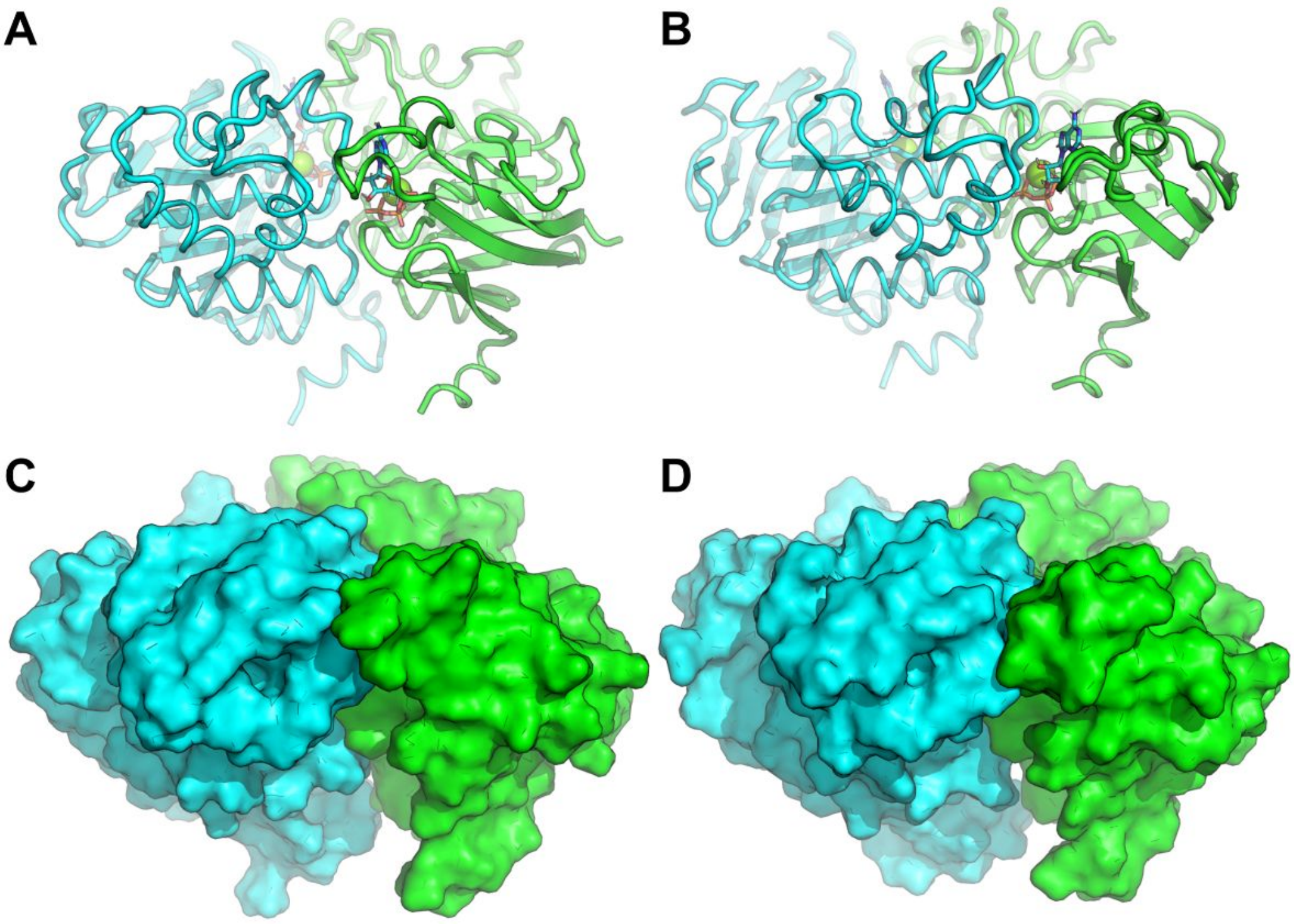

Figure S1. Structural comparison of the MD representative structure describing the wild-type NBD1/NBD2 heterodimer (panels A, C) and the cys-less NBD1/NBD2 heterodimer mutant (panels B, D). Panels A, B: cartoon representation of the MD representative structures; panels C, D: surface representation of the MD representative structures. NBD1 is colored in green, NBD2 is colored in cyan; in A and B, ATP is shown as sticks, Mg^{2+} ions are shown as green spheres. In all panels, the NBD1/NBD2 heterodimer has the same orientation due to backbone alignment (RMSD = 1.38 Å).

Mutant 3

E1329C/V1379C cys-less/V510A WT-CFTR

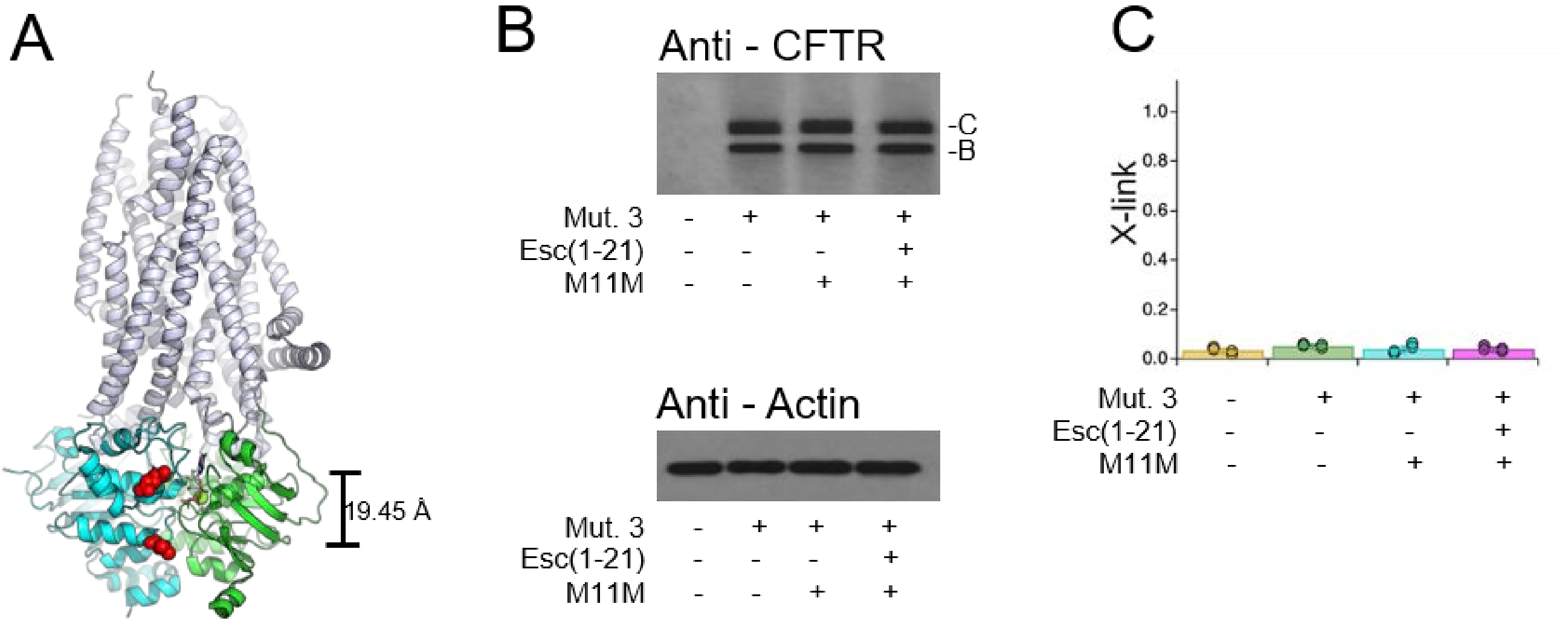

Figure S2. Lack of detectable disulfide cross-linking in CFTR double-cys mutant 3. Panel A: MD-based structural model of the cys-less/V510A WT-CFTR backbone showing the positions of the reintroduced cys residues in mutant 3 (E1329C/V1379C, red). Panel B: HEK-t cells expressing mutant 3 were incubated with (+) or without (–) 10 µM Esc(1–21) for 15 min and then treated (+) or not (–) with 0.1 mM M11M cross-linker for 20 min. Lysates were analysed by immunoblotting using anti-CFTR (upper blot) and anti-actin (lower blot) antibodies. Immature (B) and mature (C) CFTR species are indicated. Panel C: Bar graph showing quantification of the signal at the expected position of the cross linked CFTR band, normalized to actin. Data are expressed as mean ± SEM from independent experiments, with overlaid data points indicating individual measurements. Since cross-linker treatment did not generate a measurable X-link signal, no statistical analysis for Esc(1–21)-dependent inhibition of cross-linking was performed.

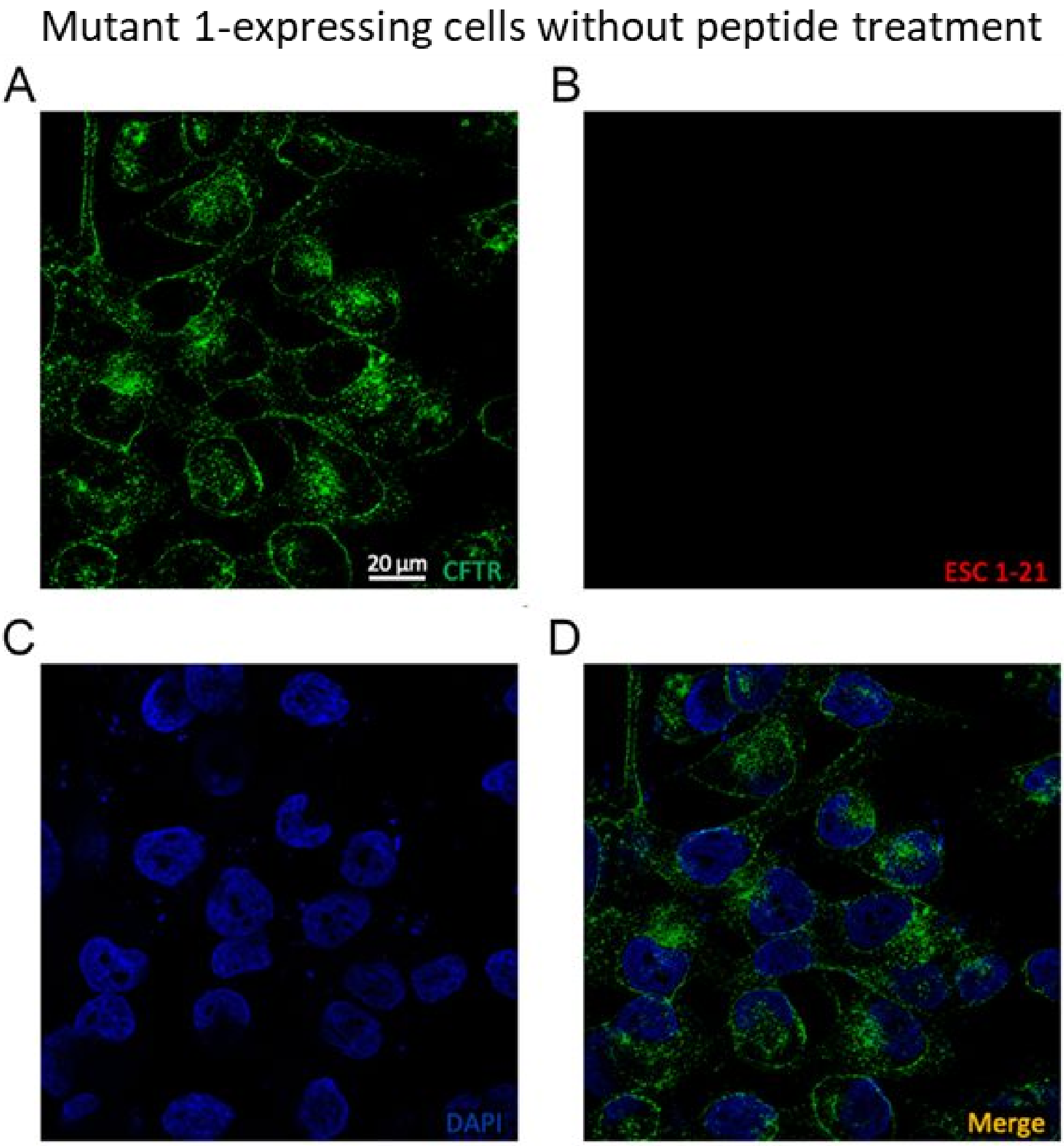

Figure S3. Confocal images of HEK-t cells expressing the T438C/E621C cys-less/V510A WT-CFTR construct. Panel A: representative confocal images of HEK-t cells expressing the construct detected with a monoclonal anti-CFTR antibody (green). Panel B: Negative control for peptide detection. Cells were not incubated with Esc(1-21) and showed no signal after staining with an anti-Esc(1-21) peptide primary antibody and an Alexa Fluor 568-conjugated secondary antibody. Panel C: Nuclear counterstaining with DAPI (blue). Panel D: Merged image showing the subcellular distribution of the cys-less CFTR mutant. Scale bar, 20 µm.

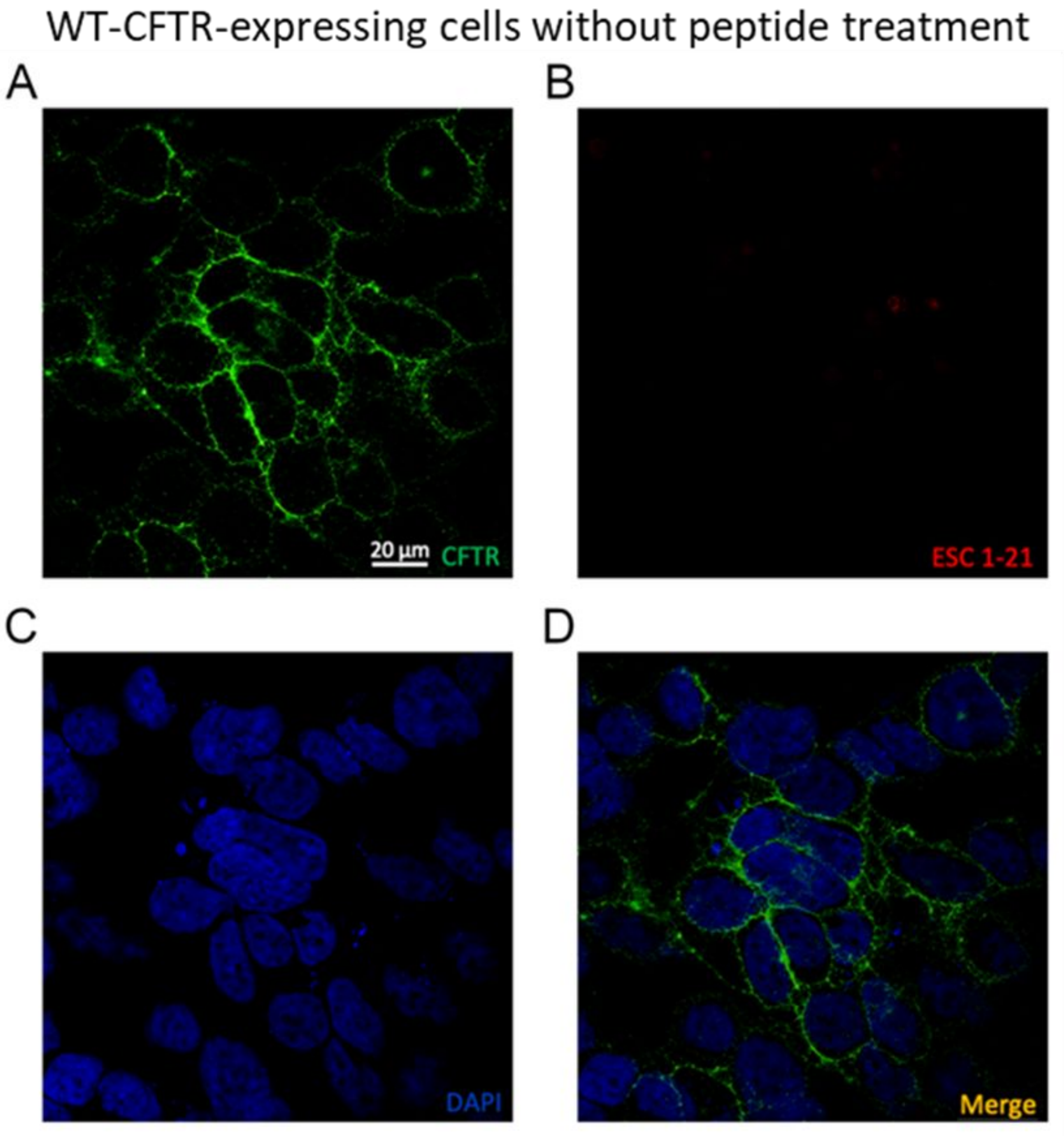

Figure S4. Confocal immunofluorescence analysis of WT-CFTR. Panel A: Representative confocal images of HEK-t cells expressing the WT-CFTR construct, detected with a monoclonal anti-CFTR antibody (green). Panel B: Negative control for peptide detection. Cells were not incubated with Esc(1-21) and showed no signal after staining with an anti-peptide primary antibody and an Alexa Fluor 568-conjugated secondary antibody. Panel C: Nuclear counterstaining with DAPI (blue). Panel D: Merged image showing the subcellular distribution of WT CFTR. Scale bar, 20 μm.

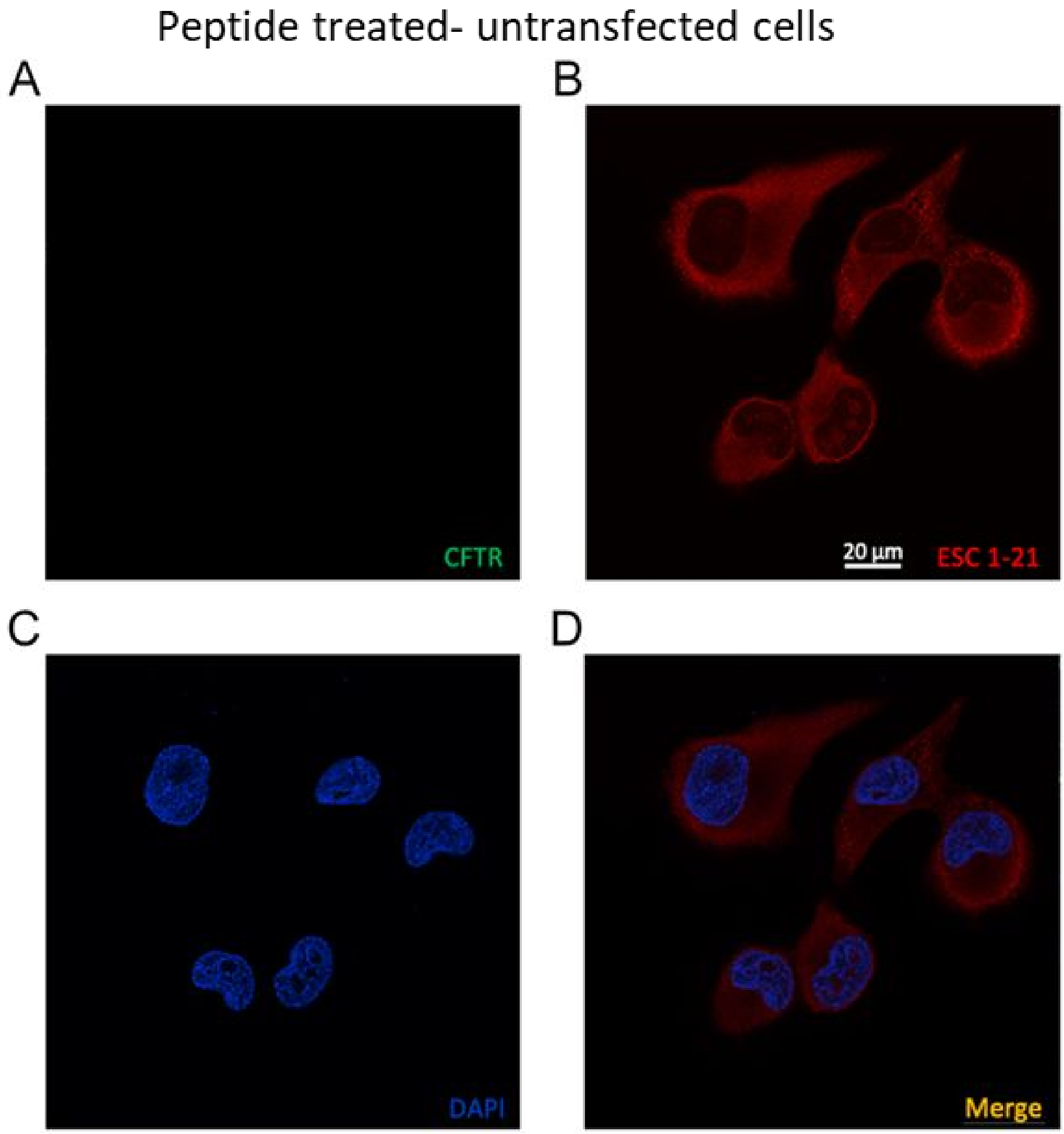

Figure S5. Confocal immunofluorescence analysis of Esc(1-21) in untransfected HEK-t cells. Panel A: Absence of CFTR signal after staining with a monoclonal anti-CFTR antibody (green). Panel B: Detection of Esc(1-21) using an anti-Esc(1-21) peptide primary antibody and an Alexa Fluor 568-conjugated secondary antibody (red). Panel C: Nuclear counterstaining with DAPI (blue). Panel D: Merged image showing the intracellular distribution of Esc(1-21). Scale bar, 20 μm.

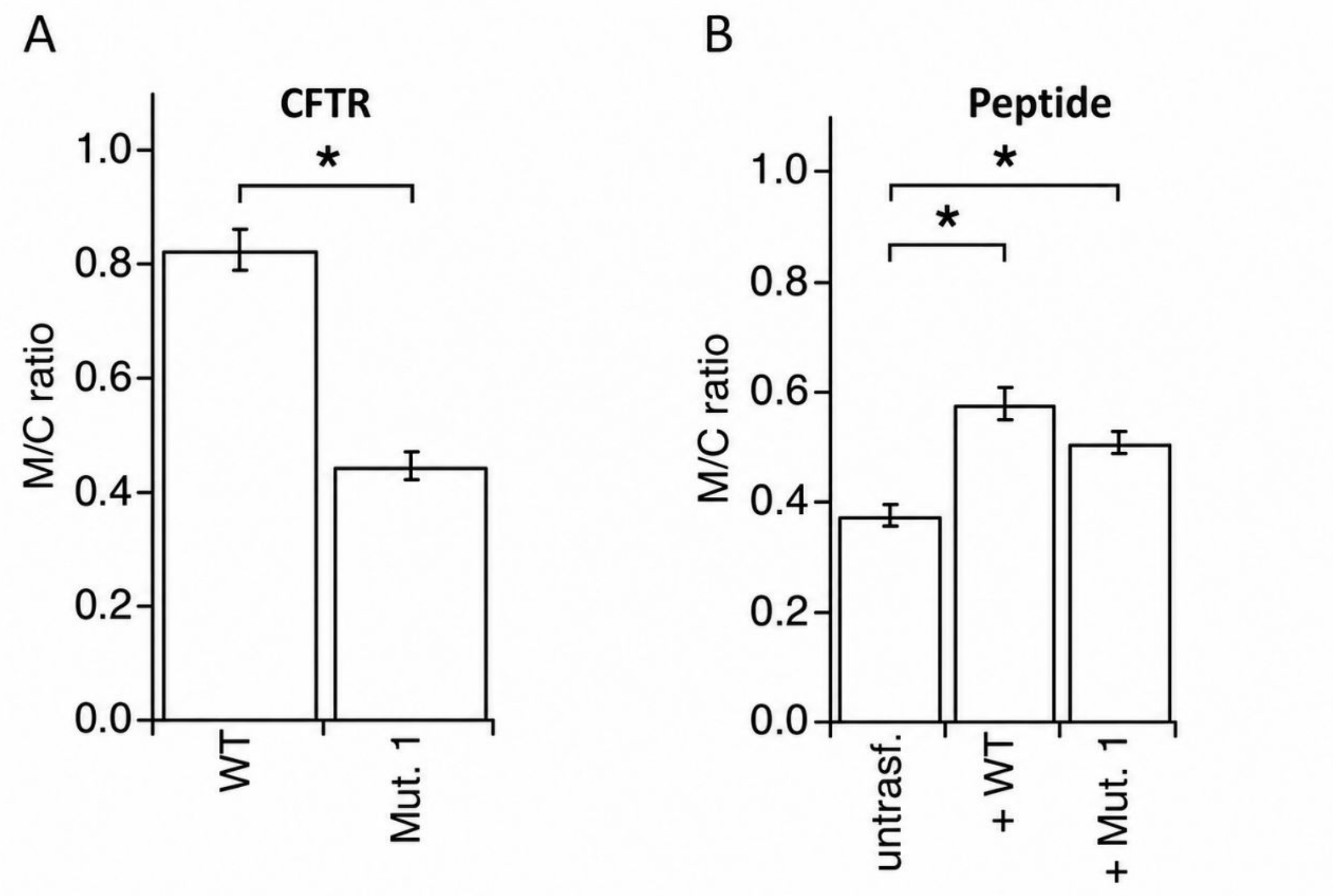

Figure S6. Quantification of membrane-to-cytosol fluorescence intensity ratios (M/C ratio). Panel A: M/C ratio of WT-CFTR and T438C/E621C cys-less/V510A WT-CFTR expressed in HEK-t cells. Panel B: M/C ratio of Esc(1-21) in untransfected cells and in cells expressing WT-CFTR or the cys-less mutant. Fluorescence intensities were measured in membrane and cytosolic regions of interest and expressed as mean ± SEM. At least 30 cells per condition were analysed. Brackets indicate the groups compared in the statistical analysis. Statistical significance was assessed using a paired two-tailed Student's t-test. Asterisks indicate statistically significant differences ($p < 0.05$) relative to WT-CFTR in panel A and to untransfected HEK-t cells in panel B.
